# Sonochemical Fabrication of Enantioselective PVDF Membranes Coated with Chiral Polymeric Nanoparticles

**DOI:** 10.3390/polym18080942

**Published:** 2026-04-12

**Authors:** Yarden Ben Moshe, Meir Abuaf, Yitzhak Mastai

**Affiliations:** Department of Chemistry, Bar-Ilan University, Ramat-Gan 5290002, Israel; yarden.silverstein@biu.ac.il (Y.B.M.); meir.abuaf@biu.ac.il (M.A.)

**Keywords:** chiral polymeric membranes, enantioselective separation, phenylalanine-based polymers, enantioselective transport, sonochemical deposition, chiral crystallization, PVDF membranes

## Abstract

Chiral polymeric nanoparticles derived from protected L/D-Phe-OMe- and unprotected L/D-Phe-based monomers were developed as functional chiral coatings for PVDF membranes to induce enantioselective recognition. The present study introduced a sonochemichal-assisted approach to the deposition of Phe-based polymeric nanoparticles onto PVDF membranes, generating chiral membrane surfaces that can facilitate enantioselective transport and crystallization. The enantioselective performance of the modified membranes was evaluated through membrane transport experiments using DL-leucine and a crystallization investigation with DL-tyrosine. Enantioselective transport experiments showed pronounced chiral resolution, achieving an enantiomeric excess (ee) of 79/76% for D/L-Leu. Furthermore, enantioselective crystallization was demonstrated using DL-tyrosine in the presence of L/D-Phe-OMe-coated membranes. Optical activity measurements, supported by SEM and DSC analysis, confirm membrane-induced enantiomeric enrichment yielding an ee of 60/68% for L/D-Tyr. These results highlight the potential of chiral polymer-coated PVDF membranes as versatile platforms for enantioselective separation.

## 1. Introduction

Chirality is a property of molecular asymmetry, defined by the inability of a molecule to be superimposed on its mirror image [1,2]. This phenomenon is widely observed in nature and plays a fundamental role in many biological macromolecules such as proteins, polysaccharides, nucleic acids and enzymes [3].

Chirality is a fundamental feature in molecular recognition processes; thus, understanding it is highly significant in many disciplines including medicine, biology, and chemistry [4,5]. Efficiently producing and separating chiral compounds is crucial for pharmaceutical development since enantiomers of the same molecule may exhibit significantly different pharmacological activity, metabolic behavior or toxicological effects [6].

Despite the significance progress in asymmetric synthesis, many pharmaceutical compounds are still manufactured as racemic mixtures, requiring efficient techniques of chiral resolution [7]. Enantioselective membrane-based separation has emerged as a promising alternative, providing high throughput, scalability, low energy requirements, and cost-effectiveness [8,9]. Enantioselective membrane-based separation can be performed using a variety of materials, including traditional polymer membranes, composite membranes with chiral selectors (e.g., CD, MOF, graphene) [10,11], and molecularly imprinted membranes (MIMs) [12,13], which are widely used for the separation of racemic mixtures [14]. Despite the growing number of new types of enantioselective membranes, many suffer from low stability, challenging manufacture, or the restricted accessibility of chiral recognition sites within the membrane matrix. In contrast, membranes with chiral surface coatings can improve enantioselective performance by exposing stereospecific functional groups directly on the membrane surface, while the underlying support provides excellent mechanical stability and scalability for practical separation processes [15,16]. Wang et al. suggested an innovative method by introducing layers of chiral super-cross-linked polymers (CHCPs on porous SiO_2_ substrates), producing chiral covalent organic polymer composite membranes. These membranes attained a remarkable ee of 91.2% for phenylalanine separation [17]. Other fascinating membranes were developed by Wang et al., who employed the phase inversion method to fabricate poly(vinylidene fluoride) (PVDF) membranes blended with chiral mesoporous SiO_2_ and β-CD, significantly enhancing hydrophilicity and enantioselectivity, achieving 55% *ee* for DL-tryptophan [18]. Chiral nano-polymeric particles have great potential in enantioselective membrane design due to their large surface area and flexible functional groups, which improve the accessibility of chiral recognition sites [19]. Over the past decade, there has been abundant discussion and research on nanoparticle-based enantiomeric recognition and chiral separation. Studies demonstrate chiral-modified nanomaterials for catalysis [20], drug separation [21], and sensing [22]. Our group developed chiral polymeric nanoparticles with controlled size distribution and modified characteristics by synthesizing a range of chiral functional polymers based on amino acids by precipitation or miniemulsion [23,24,25,26]. Here, we report the development of novel membranes functionalized with two distinct types of chiral polymeric nanoparticles based on N-acryloyl-L/D-Phe-OMe [19] (protected) and N-acryloyl-L/D-Phe-OH (unprotected) derivatives; the incorporation of these chiral polymers imparts enantioselective properties to the inherently nonselective PVDF matrix, effectively creating chiral recognition sites within the membrane structure. A sonochemical-assisted technique [27,28,29,30] was used to deposit the nanoparticles onto commercial hydrophilic PVDF membranes, which induces uniform distribution and robust adherence on the membrane surface. By integrating their superior mechanical stability, chemical resistance, and scalability [31,32] with the high density and accessibility of stereospecific sites offered by the chiral polymeric beads, these membranes present a promising platform for enantiomeric separation applications. The sonochemical-assisted technique makes the fabrication process easy and cost-effective [33]. Notably, the Phe-based nanoparticles reported here serve as a compelling proof of concept for a highly versatile functionalization platform. The modular nature of this approach facilitates the incorporation of a diverse array of chiral nanoparticles, paving the way for next-generation membranes tailored for diverse molecular recognition and selective separation challenges. The morphology of the membranes was characterized along with their chiral separation performance of racemic mixtures by enantioselective membrane transport and crystallization experiments.

## 2. Materials and Methods

### 2.1. Materials

All analytical-grade chemicals were purchased from Sigma-Aldrich (St. Louis, MO, USA) and used without further purification, including D/L-Phe methyl ester hydrochloride, triethylamine, acryloyl chloride, ethanol absolute, ethyl acetate, n-hexane (99%), azobis-isobutyronitrile (AIBN, 98%), dichloromethane (DCM), sodium bisulfate, sodium carbonate, sodium chloride, isopropanol (IPA), polyvinylpyrrolidone (PVP, 60 kDa), magnesium sulfate, DL-Tyr, DL-Leu, and tetrahydrofuran (THF). Deionized water was purified by passage through an elastane spectrum reverse osmosis system (Elga, High Wycombe, UK).

### 2.2. Synthesis of D/L-Phe-OMe Monomers

The monomers were obtained following our previously reported procedure [25]. L/D-Phe methyl ester hydrochloride (20 mmol) was placed in a 250 L round-bottom flask. DCM (90 mL) and 2.2 eq of triethylamine (4.44 g, 6.1 mL) were added, and the mixture was stirred to full dissolution and allowed to cool in an ice bath (to 0–5 °C). Acryloyl chloride (1.8 mL, 1.1 eq) was dissolved in 15 mL of DCM and added dropwise over 1 h while keeping the temperature at 0–5 °C. The mixture was stirred overnight at RT, and the clear DCM solvent was evaporated to dryness, giving a white crude product. Ethyl acetate (100 mL) was added, and after the solution was shaken thoroughly, the mixture was filtered under vacuum, and the obtained solid (HCl salts) was washed with ethyl acetate (20 mL). The ethyl acetate phase was washed with 20 mL of 1 M NaHSO_4_ (three times), 5% NaHCO_3_ and saturated NaCl (twice each). After washing, the solution was dried with MgSO_4_ and filtered. The solvent was removed under vacuum by rotary evaporation to produce the monomers (70% yield).

### 2.3. Synthesis of Protected Polymers

In a typical procedure, a solution containing PVP surfactant (0.1 g), AIBN initiator (20 mg), monomer (4 mmol, 0.8 g), and solvent (10 mL, water/2-propanol) was prepared in a 20 mL vial and deoxygenated by nitrogen purging. The sealed vial was transferred to a shaking incubator preheated to 73 °C, and polymerization continued for 24 h, after which the reaction was quenched by cooling to room temperature (RT). The formed insoluble nanospheres were separated by centrifugation and washed extensively with water and ethanol. Residual ethanol was removed by repeated centrifugation [34].

### 2.4. Hydrolysis Procedure

Protected N-acryloyl-L/D-Phe nanospheres (170 mg) were suspended in 15 mL of 1 M NaOH, followed by the addition of 2 mL THF. The mixture was sonicated for 60 s and then heated to 70 °C for ca. 4 h. The resulting clear solution was dialyzed extensively (3.5 kDa dialysis membrane) against water for 24–48 h until the pH stabilized near 7.0. The polymer solution was subsequently acidified to pH 2.0 by adding 1 M HCl, inducing the precipitation of the hydrolyzed polymers. The precipitate was washed thoroughly with water and lyophilized.

### 2.5. Polymer Deposition onto the Membrane Surface

A total of 350 mg (150 mg of unprotected polymers after hydrolysis) of poly(N-acryloyl-L/D-Phe) particles (~600 nm) was dispersed in 60 mL deionized water using bath sonication at 40 °C for 1 h, followed by probe ultrasonication for 4 min at 40% amplitude to disaggregate the particles. PVDF membranes (Durapore^®^ hydrophilic PVDF membrane, Burlington, MA, USA, 0.45 µm pore size, 47 mm diameter) were fixed in the vessel containing the polymer dispersion and irradiated for 20 min at 30% amplitude with an ultrasonic horn in an ice bath. The membranes were then washed with deionized water and dried at RT.

### 2.6. Enantioselective Membrane Transport

A stainless steel membrane filter holder (Millipore, Bedford, MA, USA and Catalog number XX3001200) with a 13 mm diameter was employed, equipped with one inlet and one outlet. An effective membrane area composed of three membrane layers (1.0 cm^2^ each) was placed inside the holder. A total of 20 mL of a 38 mM racemic leucine solution was placed in a 20 mL syringe and connected to the inlet port with a silicon tube. The syringe was placed in a syringe pump. The racemic solution was injected into the filter unit at a rate of 1 mL per hour. Samples (1 mL) were taken every hour from the vial, which was connected to the outlet port with a silicon tube. All filtration experiments were performed under identical conditions and were repeated independently twice (n = 2). All experiments were done at RT. D and L isomers were determined by High-Performance Liquid Chromatography (HPLC) measurements (Jasco PU1580, Easton, MD, USA), equipped with a UV detector (Jasco UV1570) employing a chirobiotic TAG column.

### 2.7. Enantioselective Crystallization Experiments

A solution of DL-tyrosine (30 mg/50 mL) was prepared by heating it to 65 °C until fully dissolved, cooled to RT and filtered through a 0.22 μm membrane. Two crystallization experiments were conducted, each using 15 mL of the solution. In one experiment, small pieces (~4 × 4 mm) of a chiral membrane coated with L-Phe-OMe beads were added, and in the other, small pieces of a D-Phe-OMe bead-coated membrane were used. Both setups were shaken in an ice bath (0–5 °C) for 24 h to induce crystallization. The crystallization solutions were filtered with a 0.22 μm filter, and the optical rotations of the filtrated solutions were measured by a Jasco P-2000 polarimeter using a sodium lamp light source at 589 nm passed through an 8 mm aperture. The enantiomeric excess was calculated according to the following:

ee (%) = ([R] − [S])/([R] + [S]) × 100 = %R − %S

where ee is the enantiomeric excess, and [R] and [S] are the enantiomer concentrations.

### 2.8. Characterization Techniques

Nuclear Magnetic Resonance (NMR) spectra were obtained by a Bruker DZH 400/54,400 MHz spectrometer (Bremen, Germany), and Fourier Transform Infrared (FTIR) Spectroscopy was conducted using a Thermo Scientific Nicolet 1S10 FTIR spectrometer (Hemel Hempstead, UK) equipped with a Smart iTR attenuated total reflectance sampler containing a single-bounce diamond crystal. Dynamic Light Scattering (DLS) measurements were carried out using a Zetasizer 3000 HAS (Malvern Instruments Ltd., Malvern, UK) equipped with a 4 mW He–Ne laser (λ = 632.8 nm), a detector set at a scattering angle of 173°, and a temperature-controlled cuvette holder. Scanning Electron Microscopy (SEM) was performed with a LEO Gemini 1530 Zeiss microscope (Oberkochen, Germany) operated at 0.6 kV. Differential Scanning Calorimetry (DSC) measurements were performed with a Mettler Toledo DSC 822e (Greifensee, Switzerland) equipped with a liquid nitrogen cooling accessory and calibrated with indium; samples were heated from 25 to 380 °C at 4 °C/min under N2 flow. High-Resolution Scanning Electron Microscopy (HR-SEM) images were taken using a field-emission FEI Helios 600 instrument (Hillsboro, OR, USA). The samples were coated with a 3 nm layer of iridium to decrease charging effects. HPLC and optical rotation measurements were carried out as described above. Measurements were conducted using Jasco PU1580, equipped with a UV detector (Jasco UV1570) employing a Chirobiotic TAG column. Optical rotation measurements were performed by using a Jasco P-2000 polarimeter with a sodium lamp light source at 589 nm passed through an 8 mm aperture.

## 3. Results

N-acryloyl-L/D-Phe methyl ester beads were synthesized by dispersion polymerization, yielding exclusively methyl ester-protected nanospheres. The unprotected N-acryloyl-L/D-Phe particles were subsequently obtained by basic hydrolysis. Each type of particle (i.e., the protected and unprotected variants) was, in a following step, dispersed in water for membrane modification. Commercial hydrophilic PVDF membranes were immersed in the chiral polymer suspensions, and ultrasonic treatment facilitated the deposition and attachment of the polymeric particles onto the membrane surface. The resulting coated membranes exhibit stable polymer coverage, allowing for the further study of their enantioselective separation performance.

**Scheme 1:**
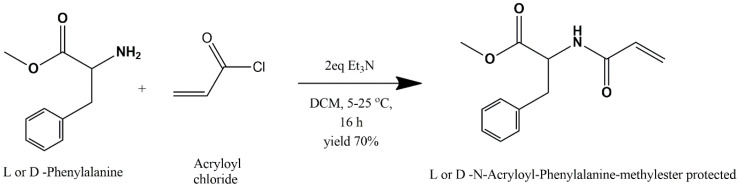
Synthesis of L/D-N-acryloyl-Phe-OMe monomers [25].

Monomer synthesis: N-acryloyl-L/D-Phe methyl ester monomers (methyl ester protective group) were synthesized in 70% yield (Scheme 1). Monomers were characterized by Mass Spectrometry (MS), 13C and 1H NMR and FTIR spectroscopy (see Supporting Information—the characterization of the monomers).

**Figure 1:**
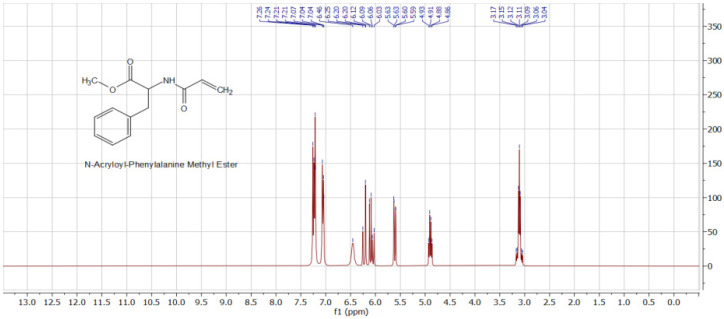
^1^H NMR spectra of L/D-N-acryloyl-Phe-OMe monomers.

The ^1^H-NMR spectrum (Figure 1) shows the transformation of primary amine to secondary amide: peaks of amide at δ 6.09 (dd, J = 18, 10 Hz, ^1^H), terminal ethylene at 5.71 (dd, J = 10, 2 Hz, ^1^H, CH_2_), 6.02 (brd, J = 8 Hz, ^1^H, CH_2_) and 6.29 (dd, J = 18, 2 Hz, ^1^H, CH) and methyl ester (δ 3.74, s, 3H). Aromatic peaks are present at δ 7.33–7.19 (m, 3H) and 7.14–7.03 (m, 2H).

The ^13^C NMR spectrum (Figure S3) further confirms the structure of the produced monomers. Characteristic carbonyl signals are observed at δ 171.9 and 164.9, corresponding to the ester and amide carbonyl carbons, respectively. The peaks of aromatic carbon appear between δ 127 and 136, while the α-carbon of the amino acid backbone appears at δ 53 and the methylene carbon at δ 38. The peak of methoxy carbon (methyl ester group) appears at δ 52.

The FTIR spectrum (Figure S4) displays specific absorption peaks at 3100–3300 and 1650 cm^−1^ corresponding to amide C=O and broad amide N-H stretching bands and 1750 and 1250 cm^−1^ (ester C=O and stretching bands). Terminal alkene stretching bond =C-H peaks are shown around 3000 cm^−1^, and aromatic C-H stretching bands are exhibited around 3300 and 2000 cm^−1^ with an evident override. The MS spectrum indicates *m*/*z* values equivalent to phenylalanine (Figure S1).

**Figure 2:**
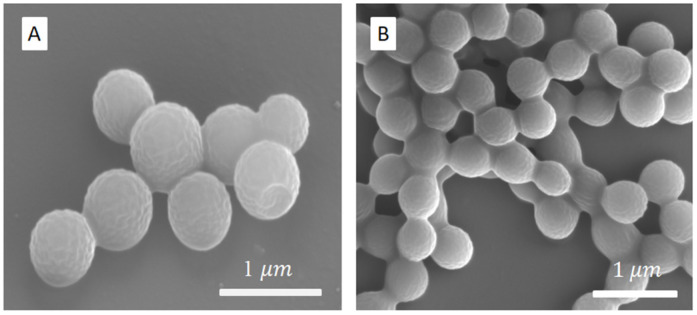
HR-SEM images of (**A**) poly-D-Phe particles precipitated in 1.2:1 water/2-propanol; (**B**) poly-D-Phe nanospheres after hydrolysis.

Polymerization: Nanospheres were synthesized by the precipitation of the dispersion polymerization of poly(N-acryloyl-L/D-Phe) in a mixture of water/2-propanol [34]. The diameter distribution of the polymer is dependent on the water/2-propanol ratio; increasing the water/2-propanol ratio leads to the higher polarity of the reaction medium and reduces the solubility of the growing poly(N-acryloyl-L/D-Phe) chains. Consequently, oligomeric radicals attain a critical precipitation length and start to nucleate to form polymeric nanospheres, resulting in a higher yield of insoluble particles [35]. A SEM image of poly (N-acryloyl-D-Phe) particles produced with a water/2-propanol ratio of 1.2 is shown in Figure 2A. Spherical polymeric particles were obtained with an average diameter of 600 nm (DLS PDI = 0.448) with a rough surface texture.

Hydrolysis: Unprotected polymers were obtained by subjecting the protected nanospheres to basic hydrolysis under alkaline aqueous conditions [36]. The THF and excess base were removed by dialysis, followed by acidification to induce polymer precipitation, forming the carboxylic acid form of the N-acryloyl-L/D-Phe nanoparticles. The particles retained their spherical morphology with an average diameter of 500 nm, as evidenced by the SEM image (Figure 2B).

**Figure 3:**
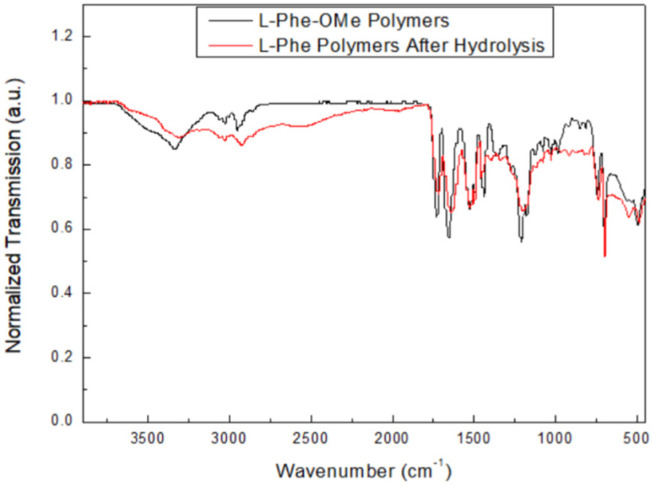
FTIR transmission spectra of poly(L-Phe-OMe) (black) and poly(L-Phe-OH) (red).

The FTIR spectrum of L-Phe-OMe polymers (black spectrum in Figure 3) reveals transmission peaks at ca. 1000–1300 and 1736 cm^−1^ corresponding to ester carbonyl stretching bands. Amide stretching bands are present at 1670 and 1530 cm^−1^. The peaks at 2800–3100 cm^−1^ correspond to aromatic and aliphatic CH, CH_2_, and CH_3_ stretching bands and that at ca. 3300 cm^−1^ to the N-H stretching band. The FTIR spectrum of the hydrolyzed polymers (red spectrum in Figure 3) displays a diminution in ester-related peaks at 1000–1300 and 1736 cm^−1^, indicating the hydrolysis of the methyl-ester protecting group. The band at ~1600 cm^−1^ was reduced relatively to the protected polymer, consistent with the formation of carboxylate groups upon hydrolysis. A clear change is also observed within the broad band peak at 2500–3300 cm^−1^, demonstrating the altered hydrogen-bonding environment in the unprotected polymer.

**Figure 4:**
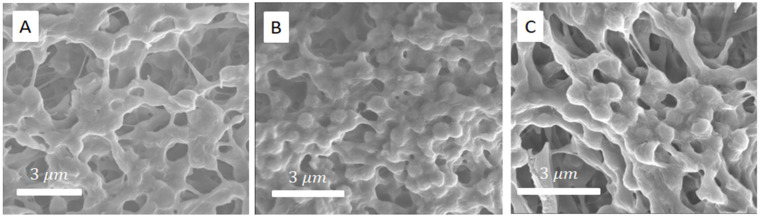
HR-SEM micrographs of three types of PVDF membranes: (**A**) pristine and coated with (**B**) unprotected poly-L-Phe beads or (**C**) poly-L-Phe-OMe particles.

Polymer deposition onto the membrane: Protected and unprotected poly(N-acryloyl-L/D-Phe) particles were deposited onto commercial hydrophilic PVDF membranes using a similar sonochemical procedure. Polymer suspensions prepared in 60 mL of deionized water were subjected to ultrasonic-assisted deposition onto the membrane, followed by rinsing with deionized water and drying. Figure 4 presents HR-SEM images depicting the surface morphology of the membranes before and after polymer deposition. Figure 4A shows the pristine membranes, characterized by a polymeric fibrous network with heterogeneous pore size. Figure 4B displays membranes with deposited unprotected poly-L-Phe beads, exhibiting a notably high density of particles on the membrane surface. The beads appear firmly attached on the surface and exhibit a broadly dispersed distribution, suggesting efficient deposition. Figure 4C shows PVDF membranes with deposited poly-L-Phe-OMe particles, presenting a robust particle loading that is slightly lower relatively to the unprotected polymer-coated membranes.

**Figure 5:**
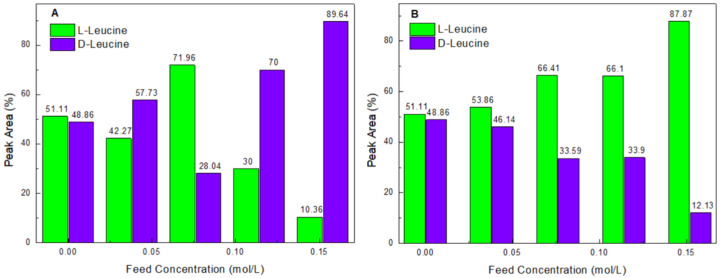
HPLC results showing peak area (%) of DL-Leu before and after filtration as function of feed concentration through (**A**) L-Phe- and (**B**) D-Phe-coated PVDF membranes.

Chiral separation performance: The enantioselective transport properties of the chiral PVDF membranes were evaluated by a chiral HPLC analysis of DL-leucine filtration. The solution was permeated through the membranes, and the enantiomeric composition of the resulting permeates was determined by HPLC and enantioselective performance ee [37]. We studied the ee of DL-Leu solutions filtered through PVDF membranes coated with unprotected poly-L/D-Phe beads using chiral HPLC. The remaining solutions were analyzed directly on a chiral column. ee was calculated relative to the initial feed, revealing the enantiomer selectivity transported by each membrane. As a reference, uncoated (commercial) PVDF membranes were used in the identical filtration experiment. An HPLC analysis of filtration through these reference membranes shows stable L/D-Leu peak areas of ca. 54/46% (±2%), corresponding to an ee of L-Leu of approximately 8%. The slight deviation from the expected 50/50 enantiomeric ratio observed for pristine PVDF membranes (54/46% ± 2%) is attributed to minor experimental uncertainties associated with the filtration procedure and HPLC analysis rather than intrinsic enantioselectivity. Since PVDF itself is an achiral polymer, no preferential interaction with either enantiomer is expected, and the observed variation falls within the typical analytical uncertainty of the measurement. Selected filtration measurements were independently repeated under identical experimental conditions (n = 2) to verify the reproducibility of the enantioselective transport studies. The reported values represent the average of independent measurements, and error bars correspond to the standard deviation of the replicates. The variability between measurements was typically within ±3%. The HPLC results for filtration through unprotected L-Phe-coated membranes (Figure 5A) exhibit a clear enantioselective transport behavior, with higher ee values in favor of D-Leu as feed concentration increased, reaching a maximum of ca. 79%. One data point at 0.076 M deviates from this general trend, showing ca. 43% ee for L-Leu. Conversely, filtration through unprotected D-Phe-coated membranes (Figure 5B) resulted in enantioselective transport favoring L-Leu, with ee values increasing up to ca. 76% at a feed concentration of 0.15M. Overall, the unprotected L-Phe-coated membranes exhibit a preferential retention of L-Leu, leading to enantioselective transport in favor of D-Leu in the permeate solution. In contrast, unprotected D-Phe-coated membranes favorably retain D-Leu, resulting in an enrichment in L-Leu in the permeate solution.

The observed enantioselective transport is likely governed by preferential interactions between the chiral nanoparticle-modified membrane surface and the enantiomers. These interactions may involve hydrogen bonding, π−π interactions, and other weak noncovalent forces, leading to the preferential adsorption of one enantiomer at the membrane interface followed by selective transport. Further mechanism studies, such as adsorption isotherms or calorimetric measurements, would be required to quantitatively elucidate the interaction mechanism.

Enantioselective crystallization: To demonstrate the chiral discrimination performance of the L/D-Phe-OMe-coated membranes, a model system based on chiral crystallization was employed. Chiral molecules typically crystallize in one of three forms: racemic compounds, in which equal amounts of left- and right-handed enantiomers are incorporated into a single crystal lattice; conglomerates, composed of physically separate crystals of each pure enantiomer; and solid solutions, where both enantiomers are randomly distributed within the crystal lattice over a defined composition range. In racemic compounds, the enantiomers share the same crystalline structure, making their separation by conventional crystallization methods more challenging than in conglomerate systems [38]. Supersaturated DL-tyrosine was chosen as an appropriate model system for enantiomeric separation by crystallization under controlled conditions. The stereoselective behavior of the protected L/D-Phe-coated membranes was demonstrated through enantioselective crystallization on their surface. Two crystallization experiments of DL-Tyr (30 mg/50 mL) were performed in the presence of either L- or D-Phe-OMe-coated membranes. Solutions were shaken in an ice bath for 24 h, after which the formed crystals were collected, dissolved in 1M HCl, and analyzed by polarimetry. Optical rotation measurements revealed enantiomeric enrichment: crystals obtained in the presence of L-Phe-OMe-coated membranes exhibited a negative optical rotation (α=−0.0311), corresponding to an ee of 60% in favor of L-Tyr, whereas crystallization on D-Phe-OMe-coated membranes yielded crystals with a positive optical rotation (α=+0.0226), corresponding to a 67.5% ee in favor of D-Tyr. The polarimetry of the dissolved crystals and ee analysis of the crystallization solutions consistently indicate membrane-induced enantioselective crystallization.

**Figure 6:**
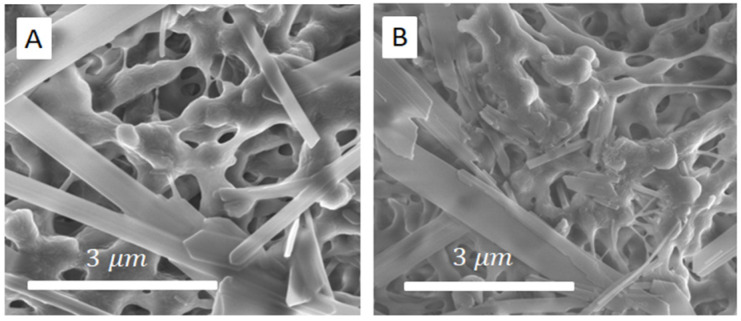
HR-SEM images of PVDF membranes (**A**) coated with poly-D-Phe-OMe particles (**B**) after crystallization from DL-Tyr solution.

**Figure 7:**
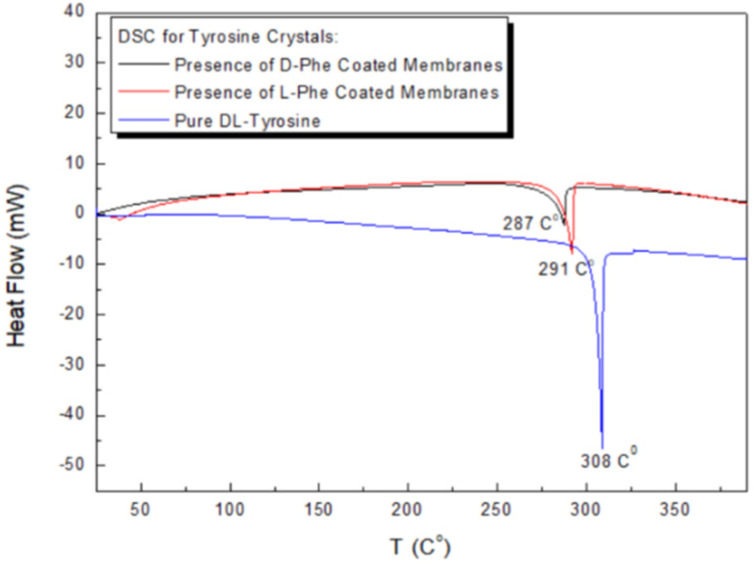
DSC results for DL-Tyr crystals: pure (blue) crystallized in presence of membranes coated with D-Phe-OMe (black) or L-Phe-OMe (red).

Figure 6 displays SEM images of membranes coated with poly-D-Phe-OMe particles after 24 h in the DL-Tyr crystallization solution. These images present needle-like tyrosine crystals on the surface of the chiral membranes (Figure 6A,B). Figure 6 demonstrates the extensive nucleation and growth of tyrosine crystals on the chiral membrane surface, accompanied by crystal formation within the solution. The control crystallization experiment performed in the absence of chiral membranes yielded DL-Tyr crystals with the characteristic racemic morphology, as confirmed in the HR-SEM image (Figure S6). No significant differences in crystal shape or surface features were observed, indicating that the chiral membranes influence enantioselective nucleation rather than crystal morphology. To examine enantioselective crystallization on the chiral membranes, we utilized DSC, as racemates and pure enantiomers differ in thermal behavior. Here, we demonstrate that the obtained tyrosine crystals exhibit properties characteristic of enantiomerically enriched crystals rather than a racemic phase [39]. For instance, previous DSC studies on histidine revealed a shift in the melting peak between racemic crystals and enantiomerically pure forms [40]. The solid samples obtained from the crystallization experiments were analyzed by DSC, revealing the chiral characteristics of the crystals. The presence of a small fraction of pure enantiomers can reduce the melting point of racemic compounds. Figure 7 shows the DSC curves of pure DL-Tyr crystals and DL-Tyr obtained from crystallization on L/D-Phe-OMe-coated membranes. DSC analysis reveals that pure racemic DL-Tyr melts at 308 °C (blue curve), whereas crystallization on chiral membranes shifts the melting point. The DSC results of DL-Tyr crystallized on L-Phe-OMe-coated membranes indicate a melting point of 291 °C (red curve), while D-Phe-OMe-coated membranes yield a melting point of 287 °C (black curve). The observed shifts in the melting point indicate enantiomeric enrichment induced by the chiral membrane’s surface.

Figure S7 displays the XRD patterns of the crystals obtained using the functionalized membranes (pink and blue), which overlap with the diffraction profile of the racemic DL-Tyr reference (black). The observed decrease in melting temperature relative to racemic DL-tyrosine may indicate partial enantiomeric enrichment or preferential nucleation induced by the chiral membrane surface, while the overall crystal structure remains largely consistent with the racemic phase, as indicated by XRD. This behavior is consistent with a conglomerate-like crystallization pathway induced by the stereospecific surface, where the membrane templates the separation of the L and D phases during the early stages of crystal growth. These results collectively demonstrate that the integration of chiral polymeric beads into the PVDF matrix imparts robust enantioselective recognition, effectively guiding the crystallization of a preferred enantiomer from racemic solutions.

## 4. Discussion

Chiral nanoparticles were prepared based on two phenylalanine-derived monomers, differing by the presence or absence of a methyl ester protecting group and their subsequent use as chiral coatings for PVDF membranes. The chiral membranes were fabricated using a sonochemical-assisted approach. This study aimed to elucidate the effect of coatings based on phenylalanine beads on the chiral recognition performance of the membranes. We hypothesized that chiral polymers bearing different Phe functionalities, including protected and unprotected forms, would enhance chiral recognition in both enantioselective crystallization and transport resolution. The enantioselective transport performance of unprotected L/D-Phe-coated membranes was evaluated using DL-leucine solutions, with chiral HPLC analysis revealing effective enantiomeric separation. In addition, the chiral discrimination ability of protected L/D-Phe-coated membranes was demonstrated using DL-tyrosine as a model system for enantioselective crystallization. Optical activity measurements, along with SEM and DSC, reveal enantiomeric enrichment on the chiral membranes. Our results show pronounced enantioselective transport, with L-Phe-coated membranes yielding a maximum *ee* of ca. 79% for D-Leu and D-Phe-coated membranes reaching ca. 76% *ee* for L-Leu, reflecting opposite enantiomer retention by the membranes. The enantioselective crystallization of DL-tyrosine in the presence of protected L/D-Phe-OMe-coated membranes led to enantioselective enrichment induced by the chiral membranes, as confirmed by the polarimetry of the dissolved crystalline products. The opposite signs and values of optical rotation, corresponding to an *ee* of 60% for L-Tyr and 67.5% for D-Tyr, together with complementary DSC and SEM analysis, demonstrate stereoselective crystallization governed by the chirality of the membrane coating. After decades of research on chiral polymeric membranes, the principles governing optimal enantioselective separation remain not fully understood. Highly efficient enantioselective transport was achieved for DL-Leu, while the enantioselective crystallization of DL-Tyr further demonstrated membrane-induced chiral discrimination. Together, these results highlight the versatility of chiral polymer-coated membranes as platforms for enantioselective separation. The present study highlights the potential of chiral polymer- coated PVDF membranes as versatile platforms for enantioselective resolution via both crystallization and transport processes. The ability to tailor polymer composition and chiral functionality offers promising opportunities for developing chiral materials that can find new applications in other fields like pharmaceutical processing and enantioselective catalysis. We hope that this work will stimulate further research on chiral polymeric particles and promote their application in emerging areas of chiral chemistry. This study extends the application of chiral materials to porous PVDF membranes functionalized with chiral polymeric nanoparticles for versatile platforms including membrane transport and template-induced crystallization.

## 5. Conclusions

This study demonstrates the successful fabrication of enantioselective PVDF membranes by coating with chiral phenylalanine-based polymeric nanoparticles using a facile sonochemical approach. The developed method enables the uniform and robust deposition of nanoparticles onto the membrane surface while preserving the intrinsic porous structure and mechanical stability of the PVDF support. Both protected (OMe) and unprotected polymeric nanoparticles retained their spherical morphology and controlled size distribution following synthesis and post-treatment. Comprehensive characterization by SEM, FTIR, and DSC confirmed the successful synthesis, hydrolysis, and effective immobilization of the chiral coatings. The unprotected polymer-coated membranes exhibited pronounced enantioselective transport behavior in DL-leucine separation experiments. L-Phe-coated membranes preferentially retained L-Leu, resulting in an enrichment in D-Leu in the permeate with a maximum ee of approximately 79%. Conversely, D-Phe-coated membranes demonstrated the opposite selectivity, yielding up to ~76% ee in favor of L-Leu. These results suggest that enantioselectivity is governed by stereospecific noncovalent interactions such as hydrogen bonding and π–π interactions at the membrane interface. In addition, the enantioselective crystallization of DL-tyrosine was successfully achieved using protected (Phe-OMe) coated membranes. The chiral membrane surfaces acted as templates for the selective nucleation and growth of preferred enantiomers. Enantiomeric excess values of approximately 60% for L-Tyr and 67.5% for D-Tyr were obtained, depending on the chirality of the coating. Polarimetry, supported by SEM and DSC analyses, confirmed membrane-induced enantiomeric enrichment. The observed melting point depression in DSC measurements further supports the formation of enantiomerically enriched crystals rather than purely racemic phases. Overall, this work presents a versatile and effective platform for enantioselective separation based on chiral polymer-coated PVDF membranes. The combination of mechanical robustness and accessible chiral recognition sites offers significant advantages for practical applications. The sonochemical deposition method provides a simple, scalable, and cost-effective fabrication route. Importantly, the modular nature of this approach allows for the tuning of polymer composition and chiral functionality for targeted applications. This strategy holds strong potential for future developments in pharmaceutical separation, enantioselective catalysis, and chiral sensing technologies. In conclusion, this study highlights the potential of chiral polymeric nanoparticle functionalization as a powerful tool for advancing membrane-based enantioselective technologies.

## Data Availability

The original contributions presented in this study are included in the article/Supplementary Material. Further inquiries can be directed to the corresponding author.

## Supplementary Materials

The following supporting information can be downloaded at https://www.mdpi.com/article/10.3390/polym18080942/s1, Figure S1: Mass spectrum of monomers; Figure S2: Table of ^1^H and ^13^C NMR and MS characterization of monomers; Figure S3: ^13^C spectrum of monomers; Figure S4: FTIR absorbance spectrum of monomers; Figure S5: Scheme of polymerization; Figure S6: HR-SEM image of DL-Tyr formed under achiral crystallization conditions; Figure S7: XRD spectra of pure crystals of L-Tyr (red) and DL-Tyr (black) and DL-Tyr crystallized with membrane coated with D-Phe-OMe (blue) or L-Phe-OMe (pink).

## Abbreviations

The following abbreviations are used in this manuscript:

MIMs	Molecularly imprinted membranes
CHCPs	Chiral super-cross-linked polymers
Phe	Phenylalanine
Leu	Leucine
Tyr	Tyrosine
AIBN	Azobis-isobutyronitrile
DCM	Dichloromethane
PVP	Polyvinylpyrrolidone
OMe	Methyl ester-protected

## References

[1] Bonner W.A. (1995). Chirality and life. Orig. Life Evol. Biosph..

[2] Prelog V. (1976). Chirality in chemistry. Science.

[3] Chen W., Qiu X., Chen Y., Bai X., Liu H., Ke J., Ji Y., Chen J. (2023). Fabrication and application of chiral separation membranes: A review. Sep. Purif. Technol..

[4] Medina D.D., Goldshtein J., Margel S., Mastai Y. (2007). Enantioselective crystallization on chiral polymeric microspheres. Adv. Funct. Mater..

[5] Preiss L.C., Werber L., Fischer V., Hanif S., Landfester K., Mastai Y., Muñoz-Espí R. (2015). Amino-acid-based chiral nanoparticles for enantioselective crystallization. Adv. Mater..

[6] Rekoske J.E. (2001). Chiral separations. AIChE J..

[7] Lorenz H., Seidel-Morgenstern A. (2014). Processes to separate enantiomers. Angew. Chem. Int. Ed..

[8] Huang S., Teraguchi M., Kaneko T., Aoki T. (2024). Preparation and Enantioselective Permeation of Polymer Membranes Containing One-Handed Helical Channels. ACS Appl. Polym. Mater..

[9] Meng C., Chen Q., Li X., Liu H. (2019). Controlling covalent functionalization of graphene oxide membranes to improve enantioseparation performances. J. Membr. Sci..

[10] Ke J., Zhang Y., Zhang X., Liu Y., Ji Y., Chen J. (2020). Novel chiral composite membrane prepared via the interfacial polymerization of diethylamino-beta-cyclodextrin for the enantioseparation of chiral drugs. J. Membr. Sci..

[11] Zhu Q., Cai Z., Zhou P., Sun X., Xu J. (2023). Recent progress of membrane technology for chiral separation: A comprehensive review. Sep. Purif. Technol..

[12] Li H., Huang Q., Li D., Li S., Wu X., Wen L., Ban C. (2018). Generation of a molecular imprinted membrane by coating cellulose acetate onto a ZrO_2_-modified alumina membrane for the chiral separation of mandelic acid enantiomers. Org. Process Res. Dev..

[13] Gao B., Cui K., Li Y. (2017). Preparation of molecule imprinted membrane of single enantiomer of amino acid with an innovative strategy and study on its chiral recognition and resolution properties. J. Chem. Technol. Biotechnol..

[14] Cheng Q., Ma Q., Pei H., He S., Wang R., Guo R., Liu N., Mo Z. (2023). Enantioseparation membranes: Research status, challenges, and trends. Small.

[15] Kartsova L., Makeeva D., Davankov V. (2019). Nano-sized polymer and polymer-coated particles in electrokinetic separations. TrAC Trends Anal. Chem..

[16] Zhao Y., Zhu X., Jiang W., Liu H., Sun B. (2021). Chiral recognition for chromatography and membrane-based separations: Recent developments and future prospects. Molecules.

[17] Wang J., Wu T., Li D., Shi Z., Liu J., Zang Y., Aoki T. (2024). Novel chiral composite membranes coated by microporous hyper-crosslinked polymer for efficiently enantioselective separation. Sep. Purif. Technol..

[18] Wang T., Huang X.-x., Wu L.-g., Li C.-j., Zhu D.-f. (2022). Fabrication of PVDF-blended ultrafiltration membranes incorporated by chiral mesoporous silica for enantioseparation. J. Ind. Eng. Chem..

[19] Abuaf M., Mastai Y. (2022). Electrospinning of polymer nanofibers based on chiral polymeric nanoparticles. Polym. Adv. Technol..

[20] Oila M.J., Koskinen A.M. (2006). Chirally modified gold nanoparticles: Nanostructured chiral ligands for catalysis. Arkivoc.

[21] Sierra I., Marina M.L., Pérez-Quintanilla D., Morante-Zarcero S., Silva M. (2016). Approaches for enantioselective resolution of pharmaceuticals by miniaturised separation techniques with new chiral phases based on nanoparticles and monolithis. Electrophoresis.

[22] Farrag M., Tschurl M., Heiz U. (2013). Chiral gold and silver nanoclusters: Preparation, size selection, and chiroptical properties. Chem. Mater..

[23] Abuaf M., Das S., Mastai Y. (2023). Organocatalytic chiral polymeric nanoparticles for asymmetric aldol reaction. RSC Adv..

[24] Menahem T., Mastai Y. (2006). Chiral soluble polymers and microspheres for enantioselective crystallization. J. Polym. Sci. Part A Polym. Chem..

[25] Moshe Y.B., Abuaf M., Mordechai C., Sharoni A., Mastai Y. (2025). Enantioselective Crystallization on Chiral Hybrid Magnetic Polymeric Particles. ACS Omega.

[26] Werber L., Preiss L.C., Landfester K., Muñoz-Espí R., Mastai Y. (2015). Isothermal titration calorimetry of chiral polymeric nanoparticles. Chirality.

[27] Cintas P., Luche J.-L. (1999). Green chemistry. The sonochemical approach. Green Chem..

[28] Perelshtein I., Applerot G., Perkas N., Wehrschuetz-Sigl E., Hasmann A., Gübitz G., Gedanken A. (2009). CuO–cotton nanocomposite: Formation, morphology, and antibacterial activity. Surf. Coat. Technol..

[29] Pol V., Motiei M., Gedanken A., Calderon-Moreno J., Mastai Y. (2003). Sonochemical deposition of air-stable iron nanoparticles on monodispersed carbon spherules. Chem. Mater..

[30] Perelshtein I., Applerot G., Perkas N., Guibert G., Mikhailov S., Gedanken A. (2008). Sonochemical coating of silver nanoparticles on textile fabrics (nylon, polyester and cotton) and their antibacterial activity. Nanotechnology.

[31] Dallaev R., Pisarenko T., Sobola D., Orudzhev F., Ramazanov S., Trčka T. (2022). Brief review of PVDF properties and applications potential. Polymers.

[32] Kang G.-d., Cao Y.-m. (2014). Application and modification of poly(vinylidene fluoride) (PVDF) membranes–a review. J. Membr. Sci..

[33] Rosales Pérez A., Esquivel Escalante K. (2024). The evolution of sonochemistry: From the beginnings to novel applications. ChemPlusChem.

[34] Melamed O., Margel S. (2001). Poly(N-vinyl α-phenylalanine) microspheres: Synthesis, characterization, and use for immobilization and microencapsulation. J. Colloid Interface Sci..

[35] Bamnolker H., Margel S. (1996). Dispersion polymerization of styrene in polar solvents: Effect of reaction parameters on microsphere surface composition and surface properties, size and size distribution, and molecular weight. J. Polym. Sci. Part A Polym. Chem..

[36] Abuaf M., Mastai Y. (2020). Synthesis of Multi Amino Acid Chiral Polymeric Microparticles for Enantioselective Chemistry. Macromol. Chem. Phys..

[37] Fernandes C., Tiritan M.E., Pinto M.M. (2017). Chiral separation in preparative scale: A brief overview of membranes as tools for enantiomeric separation. Symmetry.

[38] Menahem T., Pravda M., Mastai Y. (2009). Correlation between structures of chiral polymers and their efficiency for chiral resolution by crystallization. Chirality.

[39] Li Z.J., Zell M.T., Munson E.J., Grant D.J.W. (1999). Characterization of racemic species of chiral drugs using thermal analysis, thermodynamic calculation, and structural studies. J. Pharm. Sci..

[40] Dressler D.H., Mastai Y. (2007). Enantioselective crystallization of histidine on chiral self-assembled films of cysteine. J. Colloid Interface Sci..

