# Supplementary material for: Sonochemical Fabrication of Enantioselective PVDF Membranes Coated with Chiral Polymeric Nanoparticles
Source: Polymers (Basel). 2026 Apr 12;18(8):942. doi: 10.3390/polym18080942 (PMC13119569; doi:10.3390/polym18080942)
Supplement: Supplementary file 1 [file polymers-18-00942-s001.zip › polymers-4183422-supplementary.pdf]

polymers

MDPI

Supporting Information

Sonochemical Fabrication of Enantioselective PVDF Membranes Coated with Chiral Polymeric Nanoparticles

Yarden Ben Moshe, Meir Abuaf and Yitzhak Mastai *

Department of Chemistry, Bar-Ilan University, Ramat-Gan 5290002, Israel; yarden.silverstein@biu.ac.il (Y.B.M.); meir.abuaf@biu.ac.il (M.A.)

* Correspondence: yitzhak.mastai@biu.ac.il; Tel.: +972-3-738-4053

Characterization of N-acryloyl-L-phe methyl ester monomers:

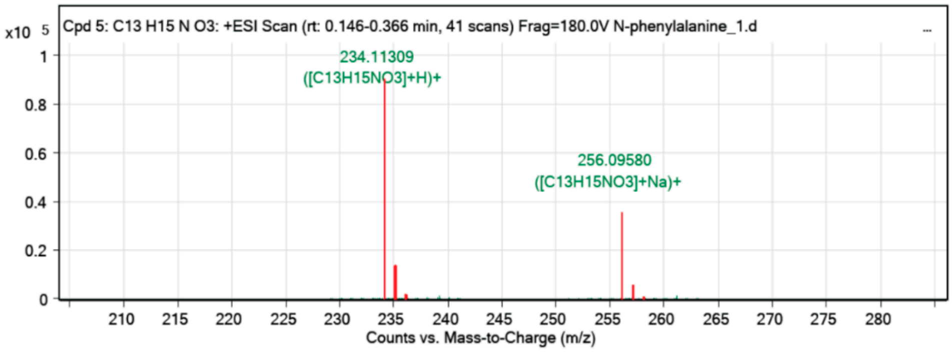

Figure S1. Mass spectrum of monomers.

Monomer	^{1}H NMR	^{13}C NMR	Mass spectroscopy
N-acryloyl-L/D-Phe methyl ester	(300 MHz, $CDCl_3$)δ: 7.33-7.19 (m, 3H), 7.14-7.03 (m, 2H), 6.29 (dd, *J*=18, 2 Hz, 1H), 6.09 (dd, *J*=18, 10 Hz, 1H), 6.02 (brd, *J*=8 Hz, 1H), 5.71 (dd, *J*=10, 2 Hz, 1H), 4.97 (dt, *J*=8, 6 Hz, 1H), 3.74 (s, 3H), abx system δ_A=3.20, δ_B=3.16 (dd, J_{AB}=13 Hz, J_{BX}= 6 Hz).	(100 MHz, $CDCl_3$) δ: 171.95 (C), 164.9 (C), 135.77 (C), 130.36 (CH), 129.32 (2xCH), 128.61 (2xCH), 127.20 (CH+CH_2), 53.18 (CH), 52.38 (CH_3) 37.88 (CH_2).	M/z (ES$^+$): 234 ([M+Na]$^+$, 100), 256 (MH$^+$, 4) 202 ([M-OMe]$^+$, 3), 174 ([M-CO_2Me]$^+$, 9).

Figure S2. Table of ^{1}H and ^{13}C NMR and MS characterization of monomers.

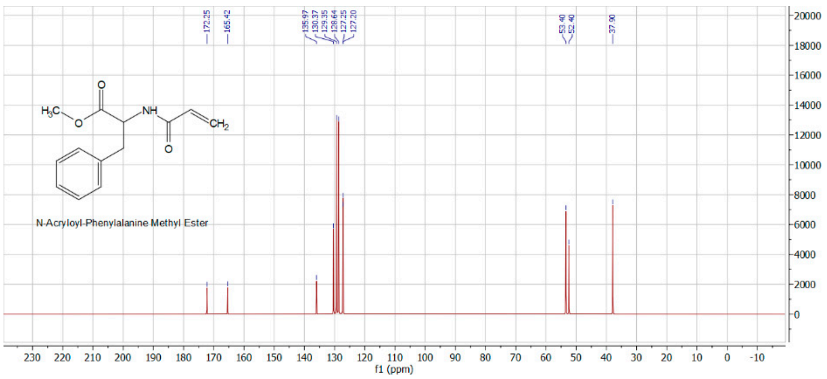

Figure S3. ^{13}C NMR spectrum of monomers.

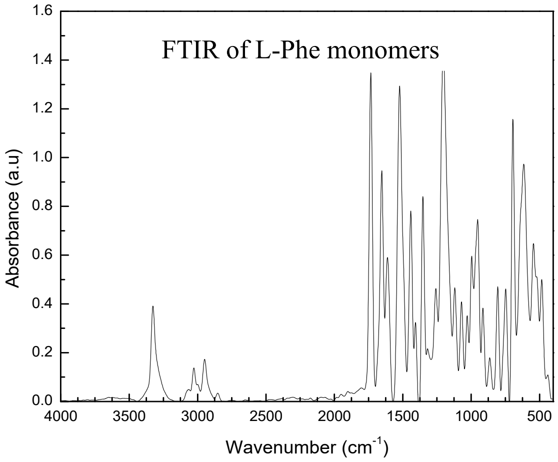

Figure S4. FTIR absorbance spectrum of monomers.

Molecular structure of monomers and polymers:

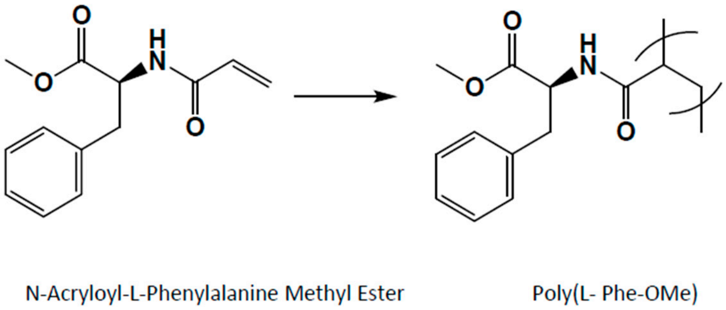

Figure S5. Scheme of polymerization.

Characterization of DL-tyrosine crystals:

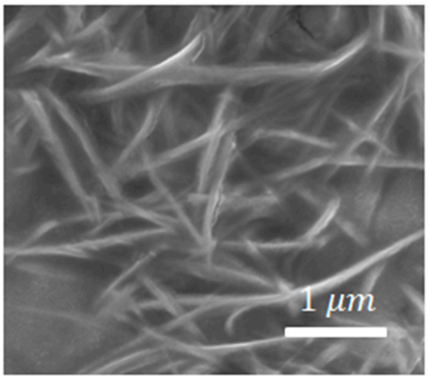

Figure S6. HR-SEM image of DL-Tyr formed under achiral crystallization conditions.

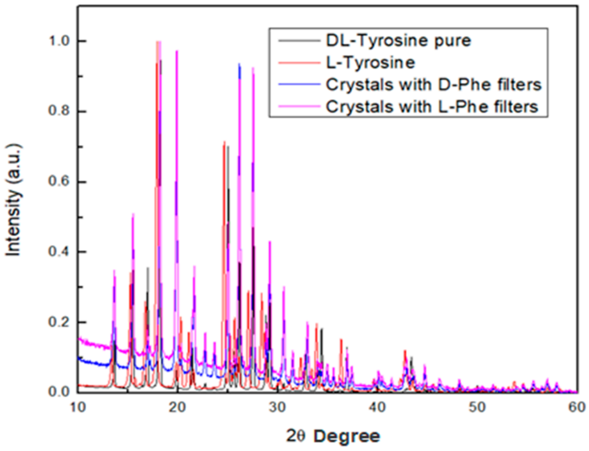

Figure S7. XRD spectra of pure crystals of L-Tyr (red) and DL-Tyr (black) and DL-Tyr crystallized with membrane coated with D-Phe-OMe (blue) or L-Phe-OMe (pink).
